# Capped antigenomic RNA transcript facilitates rescue of a plant rhabdovirus

**DOI:** 10.1186/s12985-017-0776-7

**Published:** 2017-06-13

**Authors:** Shasha Qian, Xiaolan Chen, Kai Sun, Yang Zhang, Zhenghe Li

**Affiliations:** State Key Laboratory of Rice Biology, Institute of Biotechnology, Zhejiang University, Hangzhou, 310058 China

**Keywords:** Sonchus yellow net virus, Rhabdovirus, Reverse genetics, Minireplicon, Infectious clone, Virus rescue, Hammerhead ribozyme, 35S promoter

## Abstract

**Background:**

Recovery of recombinant negative-stranded RNA viruses from cloned cDNAs is an inefficient process as multiple viral components need to be delivered into cells for reconstitution of infectious entities. Previously studies have shown that authentic viral RNA termini are essential for efficient virus rescue. However, little is known about the activity of viral RNAs processed by different strategies in supporting recovery of plant negative-stranded RNA virus.

**Methods:**

In this study, we used several versions of hammerhead ribozymes and a truncated cauliflower mosaic virus 35S promoter to generate precise 5′ termini of sonchus yellow net rhabdovirus (SYNV) antigenomic RNA (agRNA) derivatives. These agRNAs were co-expressed with the SYNV core proteins in *Nicotiana benthamiana* leaves to evaluate their efficiency in supporting fluorescent reporter gene expression from an SYNV minireplicon (MR) and rescue of full-length virus.

**Results:**

Optimization of hammerhead ribozyme cleavage activities led to improved SYNV MR reporter gene expression. Although the MR agRNA processed by the most active hammerhead variants is comparable to the capped, precisely transcribed agRNA in supporting MR activity, efficient recovery of recombinant SYNV was only achieved with capped agRNA. Further studies showed that the capped SYNV agRNA permitted transient expression of the nucleocapsid (N) protein, and an agRNA derivatives unable to express the N protein *in cis* exhibited dramatically reduced rescue efficiency.

**Conclusion:**

Our study reveals superior activity of precisely transcribed, capped SYNV agRNAs to uncapped, hammerhead ribozyme-processed agRNAs, and suggests a *cis*-acting function for the N protein expressed from the capped agRNA during recovery of SYNV from plasmids.

**Electronic supplementary material:**

The online version of this article (doi:10.1186/s12985-017-0776-7) contains supplementary material, which is available to authorized users.

## Background

The genomes of negative-stranded RNA viruses (NSVs) need to be encapsidated by nucleoprotein (N or NP) before they can be used by viral large RNA-dependent RNA polymerase (L protein) as templates for mRNA transcription and genome replication. Consequently, virions of NSVs contain nucleocapsids consisting of genomic RNAs (gRNAs) associated with core proteins. Upon entry into cells, the released nucleocapsids immediately initiate mRNA transcription for *de novo* synthesis of viral proteins and subsequent genome replication [1]. This unique mechanism of replication has significantly complicated development of strategies for recovery of NSVs from cloned cDNAs, because generation of recombinant virus entails simultaneous expression of viral RNAs and nucleocapsid core proteins in single cells for reconstitution of functional ribonucleoprotein complexes in vivo [2–4]. Another pertinent practical issue is that antigenomic RNAs (agRNAs), rather than gRNAs, have usually proven to be superior for recovery of many recombinant NSVs. This approach is thought to avoid formation of double strand RNA complexes of gRNAs and core protein mRNAs [5, 6]. Although initial breakthroughs in recovery of animal rhabdoviruses has led to refined approaches that are generally applicable to reverse genetics analysis of many NSVs, these processes are still highly inefficient, requiring optimization of various steps. Resolving these issues often involves development of a minireplicon (MR) system that consists of reporter gene(s) flanked by 5′ and 3′ noncoding regions of viral genome segments [2–4, 7]. The easily trackable MR reporter gene expression provides an ideal surrogate system to devise optimum conditions for full-length virus rescues.

Besides providing a sufficient amount of functional viral components in appropriate ratios for assembly of biologically active nucleocapsids, proper processing of viral RNA transcripts to match their authentic termini has also proven to be of critical importance for efficient recovery of NSVs [8–10]. In animal NSV rescue systems, generation of exact viral RNA 5′ termini is often achieved by positioning viral cDNAs immediately downstream of a T7 polymerase (T7 Pol) promoter or an RNA polymerase I (Pol I) promoter for precise transcription [2–4]. Both T7 Pol and Pol I are able to initiate transcription at defined position to generate uncapped transcripts matching the exact 5′ end sequence and structure of authentic viral RNAs. An autolytic hepatitis delta virus ribozyme (HDV Rz) fused to the 3′ end of viral cDNA is regularly employed to produce the exact 3′ terminus. Several studies have also reported the use of RNA polymerase II (Pol II) promoter to direct viral RNA transcription [8, 10–13]. Unlike T7 Pol and Pol I, Pol II normally produces capped, polyadenylated transcripts with variable 5′ and 3′ non-viral nucleotide extensions, which necessitates the use of both a hammerhead ribozyme (HH Rz) and an HDV Rz to produce exact viral RNA ends [8, 10–13]. Overall, these in vivo transcription systems have been used to rescue various animal NSVs that differ in genome structure (segmented or nonsegmented) and replication sites (nuclear or cytoplasmic).

*Sonchus yellow net virus* (SYNV) belongs to the genus *Nucleorhabdovirus* of the family *Rhabdoviridae*, and contains a nonsegemented genome of negative polarity. The SYNV genome encodes five structural proteins conserved in other rhabdoviruses of plants and animals, i.e. the N protein, phosphoprotein (P), L protein, matrix protein (M), and the glycoprotein (G), and an additional non-structural protein (sc4) involved in cell-to-cell movement. The N, P, and L core proteins associate with gRNAs or agRNAs to form nucleocapsids, which carry out mRNA transcription and genome replication activities. Flanking the protein-coding region in the SYNV genome are the 3′ leader and 5′ trailer sequences that contain important *cis*-elements for encapsidation and RNA synthesis [14]. The biology of SYNV has been studied in most details amongst plant rhabdoviruses, and more importantly, recently developed SYNV MR and full-length virus reverse genetics systems represent the first plant NSVs amenable to genetic manipulation [15, 16]. These SYNV systems rely on cauliflower mosaic virus (CaMV) 35S promoter-mediated Pol II transcription of SYNV agRNAs or MR agRNA derivatives, and together of the N, P, and L core protein mRNAs. In the MR system, the exact 5′ and 3′ termini of SYNV agRNA transcripts were processed by a 5′ HH Rz and a 3′ HDV Rz, respectively [15], as is the case with other Pol II-based systems used to rescue many animal NSVs [8, 10–13, 17]. However, in the full-length SYNV rescue system, viral agRNAs were precisely transcribed by a truncated 35S promoter (35St) [16], an approach commonly used to produce infectious transcripts of positive-stranded RNA viruses in plant cells [18]. The later strategy obviates the need of using an HH Rz for 5′ processing, but 5′ capped viral agRNA transcripts are generated during transcription. Although in nature both gRNAs and agRNAs of NSVs are uncapped, the capped SYNV agRNA transcripts are able to initiate recombinant infections in agroinfiltrated plants when core proteins are also co-expressed.

Although both strategies have generated functional SYNV agRNA derivatives, the template activities of the HH Rz-processed and the capped, 35St-precisely transcribed agRNAs have not been compared in parallel. In this study, we have shown that SYNV agRNAs cleaved by an optimized HH Rz variant compared to 35St capped counterparts lead to similar levels of MR reporter gene expression; however, recombinant SYNV was efficiently rescued only by using the capped agRNA transcripts. In addition, we provide evidence that the SYNV N proteins expressed from capped agRNAs facilitate the rescue process. Our study thus reveals a previously unrecognized *cis*-acting function of the N proteins expressed from agRNAs during NSV rescue processes, and highlights precautions that may need to be considered when engineering optimized conditions for MR activity versus full-length virus rescue.

## Methods

### General

Plasmid DNA was extracted using an AxyPrep plasmid miniprep kit (Axygen, Union, CA) or a QIAGEN plasmid miniprep kit (QIAGEN, Hilden, Germany) and used for cloning and sequencing. DNAs for subcloning were recovered from gel slices using an AxyPrep DNA gel extraction kit (Axygen, Union, CA) or a Purelink Quick gel extraction kit (Invitrogen, Shanghai, China). Restriction enzymes were obtained from New England BioLabs (Shanghai, China) or Thermo scientific (Hangzhou, China), and chemicals were purchased from Sigma Chemical (Shanghai, China). Oligonucleotides used for PCR amplifications were purchased from Invitrogen (Shanghai, China) or TsingKe (Hangzhou, China), and amplifications were performed using a high-fidelity KOD-Plus-Neo DNA polymerase (Toyobo, Osaka, Japan) or Phusion DNA polymerase (New England BioLabs, Shanghai, China). All clones were sequenced at Invitrogen (Shanghai, China) or TsingKe (Hangzhou, China).

### Construction of plasmids

The p35S-HH1-MR_GFP-RFP_ plasmid has been described previously by Ganesan [15] and used as a starting point to construct other vector containing modified HH Rz variants. The HH1 Rz sequence downstream of the double 35S promoter was replaced by a sequence encoding the HH2 Rz (5′-CTGTCT CTCTGATGAGGCCGAAAGGCCGAAACCCGGTATCCCGGGTC-3′), HH3 Rz (5′-CTGTCTCTCTGATGAGGCCGAAAGGCCGAAACTATAGGAAAGGAATTCCTATAGTC-3′), or HHm Rz (5′-CTGTCTCTCTGATGAGGCCGAAAGGCCGAAACCCGGTATCCCGGGTG-3′). The resulting vectors were named p35S-HH2-MR_GFP-RFP_, p35S-HH3-MR_GFP-RFP_ and p35S-HHm-MR_GFP-RFP_, respectively. The primer sequences used for plasmid constructions are shown in Additional file 1: Table S1.

To generate the p35S-HH2-MR_GFP-RFP_ plasmid, the HH2 Rz sequence was incorporated into the plasmid in three steps. First, a fragment spanning the T-DNA RB and HH1 sequence from p35S-HH1-MR_GFP-RFP_ was amplified by PCR with the primer pair T-DNA RB F/pCambia HRz R1. The PCR product was elongated further by a second round of PCR using primer pair T-DNA RB F/HRz *Xma*I R2, followed by ligation into the *Xma*I and *Hind*III sites of the vector p35S-HH1-MR_GFP-RFP_ to yield an intermediate plasmid. Finally, the region between the SYNV leader and T-DNA LB sequences was amplified from the plasmid p35S-HH1-MR_GFP-RFP_ with the primers TC+ SYNV(+)1 *Xma*I F and T-DNA LB *Spe*I *Pvu*I R, and then inserted into the above mentioned intermediate plasmid after double digestion with *Xma*I and *Sac*I. To construct the p35S-HH3-MR_GFP-RFP_ plasmid, the p35S-HH1-MR_GFP-RFP_ plasmid was used as a scaffold sequence and template. The PCR product was amplified using the primers SYNV HRz_3_ F/SYVV HRz_3_ R and assembled through an In-Fusion HD PCR Cloning kit (Clontech, Japan). To yield the p35S–HHm-SYNV MR_GFP-RFP_, the HH2 catalytic sites were mutated from GUC to GUG by mutagenesis PCR using the primers TG+ SYNV(+)1 *Xma*I F and T-DNA LB *Spe*I *Pvu*I R, the resulting HHm PCR products were digested with *Xma*I and *Sac*I and then inserted into the intermediate plasmid. The p35St-MR_GFP-RFP_, which contained a truncated 35S promoter, was generated by removing the sequence between the transcriptional initiation site of promoter and the hammerhead ribozyme of p35S-HH1-MR_GFP-RFP_. A fragment encompassing the SYNV leader and the T-DNA RB sequence from the vector p35S-HH1-MR_GFP-RFP_ was amplified by PCR with the primers SYNV(+)1 F and T-DNA LB *Spe*I *Pvu*I R. The resulting PCR product was digested with *Sac*I and ligated into the *Stu*I and *Sac*I sites of p35S-HH1-MR_GFP-RFP_ to give rise to the p35St-MR_GFP-RFP_ plasmid.

To construct the p35S-HH3-SYNV transcription plasmid, the HH3 Rz sequence was inserted into the p35St-SYNV [16] between the 35S promoter and the SYNV leader region. The fragment spanning the *Not*I site and the 5′ untranslated region (UTR) of the N gene was amplified from the p35S-HH3-MR_GFP-RFP_ plasmid using the primer pair pCB301 *Not*I F/N 5′ UTR R, and the fragment between the N gene and the *Nco*I site located in the N/P gene junction was amplified from the p35St-SYNV plasmid with the primer pair N 5′ UTR F/pCB301 *Nco*I R. The two resulting PCR fragments were inserted into the *Not*I and *Nco*I digested p35St-SYNV vector by In-fusion cloning to give rise to the target plasmid.

For construction of the p35St-SYNV-GFP_le/N_ plasmid, the GFP reporter gene was incorporated into the region between the leader sequence and the 5′ UTR of the N gene within the p35St-SYNV plasmid through three PCR reactions. The first PCR was performed using a forward primer (P 5′ UTR-N F) spanning the 5′ UTR of the P gene with a reverse primer (pCB301 *Nco*I R) containing the *Nco*I site. For the second PCR, a forward primer (pCB301 *Not*I F) encompassing the *Not*I site was used with a reverse primer (N 5′ UTR R) annealing to the 5′ UTR of the N gene. For both PCRs, the plasmid p35St-SYNV was used as the DNA template. The third PCR was performed to amplify the fragment between 5′ UTR of the N gene and the N/P junction sequence from the plasmid p35St-MR_GFP-RFP_, with the primers N 5′ UTR F and SYNV N P junction R. Following purification, the PCR products were inserted into the *Not*I/*Nco*I sites of p35St-SYNV through In-fusion cloning.

### Plant growth and *Agrobacterium* infiltration

*Nicotiana benthamiana* plants were grown in a greenhouse maintained at 23–25 °C with a 16 h day/night regimen. *Agrobacterium tumefaciens* strain EHA105 harbouring recombinant binary plasmids were grown to stable phase in LB media supplemented with appropriate antibiotics as previously described [15]. The cultures were pelleted by centrifugation at 4000 g and resuspended in MMA buffer [10 mM MES, 10 mM MgCl_2_ and 100 μM Acetosyringone, pH 5.6] to an optical density (OD) A_600_ of 0.8, followed by incubation at room temperature for 2 to 4 h. Equal volumes of *Agrobacterium* cultures harbouring the pGD:N, pGD:P, pGD:L (or pGD:NPL) and pGD:MR ribozyme derivatives (or the pCB301:SYNV infectious clone derivatives) were mixed in the desired combinations with one volume of agrobacterial mixtures containing the tomato bushy stunt virus p19, barley stripe mosaic virus γb, and tobacco etch virus P1/HC-Pro plasmids. In the case of NPL + L core protein expression conditions, one volume of pGD:NPL culture at OD_600_ of 0.8 was used to substitute for the N + P + L mixture, and an additional volume of pGD:L was supplied as described [16]. Agrobacterial mixtures were then pressure infiltrated into *N. benthamiana* leaves with a 1 mL syringe. When assessing the transcript levels or transient protein expressions for agRNA derivatives, *Agrobacteria* containing the core protein supporting plasmids were not included.

### Fluorescence microscopy and photography

Fluorescence in *N. benthamiana* infiltrated leaves was visualized with a Zeiss SteREO Lumar V12 epifluorescence microscope. The excitation and emission wavelengths are 470/40 and 525/50 nm for GFP detections, and 565/30 and 620/60 for RFP detections. The data were processed with LSM software Zen 2009 (Carl Zeiss). The symptoms in recombinant p35St-SYNV-GFP_le/N_ or p35St-SYNV-GFP_N/P_-infected leaves were also monitored under long wavelength UV light. Photographs were taken under white light and UV light.

### Analysis of proteins

Total proteins were extracted from healthy or agroinfiltrated *N. benthamiana* leaf samples and evaluated by Western blotting. Proteins separated by SDS-PAGE were transferred to nitrocellulose membranes and stained with Ponceau S, followed by detecting with polyclonal antibodies specific to the disrupted SYNV virions, N protein, or monoclonal antibodies against GFP and RFP (Abcam, Cambridge, UK).

### In vitro transcription and ribozyme cleavage assay

To produce in vitro RNA transcripts of HH Rz variants, we amplified the HH Rz cDNAs from the MR transcription constructs using the primers T7 HH MR F and N 5’UTR R. The PCR products contained the T7 promoter upstream of HH, followed by the SYNV leader sequence and the 5′ UTR of the N gene. In vitro T7 transcription was carried out in a total reaction volume of 50 μL containing 5 μg recovered PCR product, 5 μL 10 × T7 RNA polymerase transcription buffer, 0.5 μL rNTPs (each 100 mM), 10 μL DTT (50 mM), 0.1 μL RNase inhibitor (40 U/μL, Ambio, Life Technology) and 1 μL T7 RNA Polymerase (50 U/μL, TaKaRa, Japan). The mixtures were incubated at 37 °C for 2–4 h, and then 4 μL DNaseI (5 U/μL from TaKaRa, Japan) was added to remove the template DNA. The RNA transcripts were extracted by phenol/chloroform, precipitated by ethanol, and dissolved in 50 μL sterile ddH_2_O.

To analyse the in vitro HH Rz cleavage, 10 μL ribozyme-substrate transcripts were incubated in the reaction buffer [10 mM Tris–HCl (pH 8.0), 10 mM NaCl and 1 mM EDTA (pH 8.0)], and heat treated for 1 min at 80 °C, followed by snap cooling on ice. The cleavage reaction was initiated by supplementing MgCl_2_ to a final concentration of 0.5 mM. After 2 h incubation at room temperature, the reaction was terminated by addition of denaturing 2 × RNA loading buffer, heated for 2 min at 95 °C, and snap cooled on ice. The RNA samples were loaded on a 6% polyacrylamide gel containing 5 M urea to separate the reaction products, followed by staining with ethidium bromide to visualize the RNA bands.

### Semi-quantitative reverse transcription PCR

To assess the hammerhead ribozyme processing activity in vivo, *N. benthamiana* leaves infiltrated with SYNV MR containing HH Rz variants (HH1, HH2, HH3, and HHm) were collected at 5 dpi. Total RNA was extracted using the TRIzol (Invitrogen) method, and reverse transcribed with ReverTra Ace qPCR RT Master Mix kit (Toyobo). The complementary DNA (cDNA) products were used as the PCR template to amplify the desired fragments spanning the HH region under appropriate 28 cycles with the primer pair MR HR SqRT-PCR F/MR HR SqRT-PCR R (Additional file 1: Table S1). The *N. benthamiana* actin gene (*NbACTIN*) was amplified using the primer pair Actin qPCR F/Actin qPCR R (Additional file 1: Table S1) to serve as a control standard. After the PCR reaction, DNA products were resolved on an agarose gel, and the concentration of DNA bands representing the uncleaved ribozyme-substrate RNAs were quantified by ImageQuant software (GE Healthcare).

### Quantitative reverse transcription PCR (qRT-PCR) and Northern blot analyses

*N. benthamiana* leaves were infiltrated with bacteria harbouring the SYNV minireplicon plasmids (p35St-MR_GFP-RFP_ or p35S-HH3-MR_GFP-RFP_), or the infectious clone derivatives (p35St-SYNV or p35S-HH3-SYNV). RNA was isolated from leaf samples collected at 5 dpi and reverse transcribed into cDNA. qRT-PCR was performed with gene-specific primers for GFP, RFP, N, P, G and control *NbACTIN* (primers named qPCR F/R in Additional file 1: Table S1) using the cDNA as the template. For Northern hybridizations, total RNA samples were resolved in 1.2% agarose-formaldehyde gels, and probed with Digoxin-labeled GFP or RFP DNA probes. Probes used to detect GFP and RFP agRNAs were amplified with the primer pairs eGFP/F and eGFP/R and DsRed/F and DsRed/G4A/R, respectively, using the p35S-HH1-MR_GFP-RFP_ plasmid as a template.

## Results

### Differential SYNV rescue efficiency with capped or 5′ hammerhead ribozyme-processed viral agRNAs

**Fig. 1:**
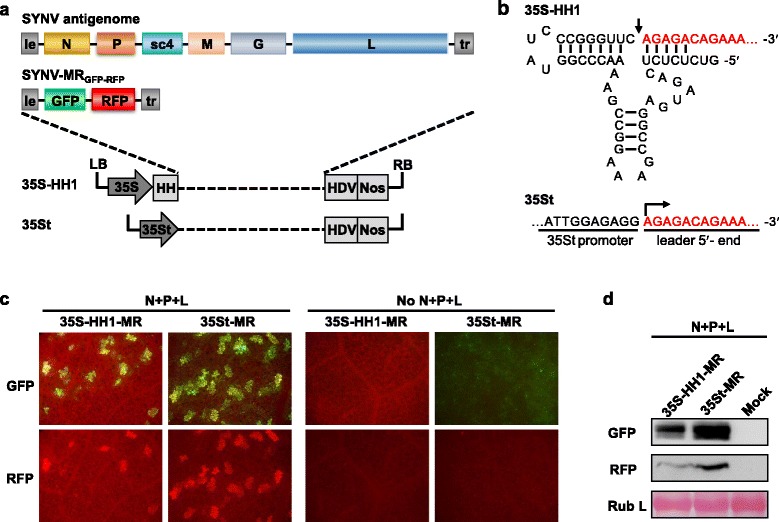
SYNV reverse genetics systems using a truncated 35S promoter and 5′ hammerhead ribozyme to process viral antigenomic (ag) RNAs. **a** Schematic representation of the SYNV full-length agRNA and a minireplicon (MR) derivative (SYNV-MR_GFP-RFP_) containing GFP and RFP genes replacing the N and P ORFs, respectively. The grey illustrations depict the cauliflower mosaic virus 35S promoter-based transcription plasmids (35S-HH1) for posttranscriptional processing of agRNAs with a hammerhead ribozyme variant (HH1) to yield precise uncapped 5′ termini, and a truncated promoter (35St) for synthesis of SYNV agRNAs with capped precise 5′ termini. Both constructs employed a hepatitis delta virus (HDV) antigenomic ribozyme followed by a NOS terminator to release the exact agRNA 3′ termini. **b** Illustration of 5′ terminus processing by HH1 Rz (upper panel) and 35St (lower panel). The sequences of the HH1 Rz and the 35St promoter are shown in black, and the 5′ termini of SYNV leader are shown in red. Arrows indicate the HH1 Rz cleavage site in 35S-HH1, or the transcription start site of the 35St. **c** Comparison of reporter gene expression mediated by 35S-HH1-MR and 35St-MR reporters in agroinfiltrated leaves. Agroinfiltration mixtures for delivery of the 35S-HH1-MR or 35St-MR transcription plasmids were infiltrated into *N. benthamiana* leaves. In the left panels, *Agrobacteria* mixtures harbour plasmids for expression of the N, P, and L core proteins (N + P + L) and viral suppressors of RNA silencing, and the right panels illustrate results of parallel experiments in which the core protein plasmids were omitted from the *Agrobacteria* mixtures (No N + P + L). Expression of fluorescent reporters in infiltrated leaf tissues at 6 days postinfiltration (dpi) was monitored by fluorescence microscopy under GFP (upper panels) and RFP (lower panels) channels. **d** Protein gel blots of GFP and RFP recovered from agroinfiltrated leaf extracts assessed by the GFP- and RFP-specific antibodies. The protein blots were stained with Ponceau S to verify similar levels of the large subunit of Rubisco (Rub L)

Generation of viral RNA transcripts with exact 5′ and 3′ termini is essential for initiation of NSV infections during reverse genetics experiments [8–10, 13, 15]. To address this issue with SYNV, we constructed two SYNV agRNA transcription plasmids based on the CaMV 35S promoter that result in different strategies for processing the viral 5′ termini. The 35S-HH1 transcription plasmid contains an autolytic HH Rz variant (HH1) positioned downstream of the 35S promoter, whereas the 35St plasmid has a truncated 35S promoter (35St), in which the 3′ end sequence downstream of transcription start site is removed (Fig 1a). Both the 35S-HH1 and 35St plasmids contain a self-cleaving HDV Rz to release the authentic viral 3′ end. To compare the template activities of viral transcripts produced by the 35S-HH1 and 35St, we cloned a cDNA fragment of a SYNV agRNA-derived minireplicon (MR_GFP-RFP_) into these plasmids. In MR_GFP-RFP_, the SYNV N and P open reading frames (ORFs) were replaced by the green and red fluorescence protein (GFP and RFP) genes, respectively, and flanked by the viral 5′ leader and 3′ trailer sequences [15] (Fig. 1a). Both the 35S-HH1 and 35St transcription plasmids were designed to produce viral RNAs of identical sequence, although in the latter case the viral transcripts were expected to be capped by the host capping machinery (Fig. 1b). *Agrobacterium* strains harbouring the 35S-HH1-MR or 35St-MR plasmid, along with necessary supporting plasmids for expression of the SYNV N, P, and L core proteins, were co-infiltrated into *N. benthamiana* leaves. In addition, *Agrobacteria* cultures carrying the plasmids coding for the tomato bushy stunt virus p19, barley stripe mosaic virus γb, and tobacco etch virus P1/HC-Pro viral suppressors of RNA silencing (hereafter referred to as VSRs) were included into the infiltration mixture to suppress host RNA silencing responses [15]. As shown in Fig. 1c, the numbers of GFP and RFP foci and their fluorescence intensities were greater in 35St-MR infiltrated leaves than those in 35S-HH1-MR leaves. In addition, Western blot analyses also indicated that 35St-MR supported higher levels of reporter protein expression than the 35S-HH1-MR infiltrations (Fig. 1d). Notably, in the absence of the N, P, and L supporting plasmids, the 35St-MR infiltrated leaf patches displayed a faint and evenly distributed low level of green fluorescence but an RFP signal was not detected, whereas neither the green nor the red fluorescence appeared in the 35S-HH1-MR infiltrated leaf tissues. These results suggest that the core protein-independent GFP expression observed in the 35St-MR infiltrated leaves likely resulted from direct translation of the capped MR agRNA transcripts, in which the GFP ORF positioned at the 5′ end of MR agRNA was readily available for ribosome scanning.

We next engineered full-length SYNV agRNA cDNA clones into the 35St and 35S-HH1 transcription plasmids to generate 35St-SYNV [16] and 35S-HH1-SYNV, respectively, and compared their resulting infectivities when supported by core proteins and VSRs co-delivered via agroinfiltration. The initial experiments were carried out using the three individual plasmids to express the N, P, and L proteins (N + P + L, Table 1). Under these conditions, approximately 4% of the 35St-SYNV infiltrated *N. benthamiana* plants became infected, whereas systemic infections were not observed amongst the plants infiltrated with 35S-HH1-SYNV.

**Table 1:** Systemic infection of SYNV antigenomic cDNA derivatives in *Nicotiana benthamiana*

agRNA clone derivatives^a^	Core proteins^b^	Infectivity (No. of infected/inoculated plants)^c^			
		Exp. 1	Exp. 2	Exp. 3	Total (percentage)
35St-SYNV	N + P + L	2/50	1/40	2/40	5/130 (3.8%)
35S-HH1-SYNV	N + P + L	0/50	0/40	0/40	0/130 (0%)
35St-SYNV	NPL + L	8/40	7/35	9/44	24/119 (20.2%)
35S-HH1-SYNV	NPL + L	0/40	0/38	0/43	0/121 (0%)
35S-HH3-SYNV	NPL + L	0/40	0/35	1/44	1/119 (0.84%)
35St-SYNV-GFP_N/P_	NPL + L	4/24	9/44	7/50	20/91 (21.9%)
35St-SYNV-GFP_le/N_	NPL + L	1/24	2/44	1/50	4/91 (4.4%)
35S-HH3-SYNV	NPL + L + N	0/36	0/36	-	0/72 (0%)

^a^SYNV antigenomic cDNA and its derivatives were cloned into the pCB301 binary vector and positioned downstream of a hammerhead (HH) ribozyme variant or directly under control of a truncated 35S promoter (35St)

^b^N: pGD-N; P: pGD-P; L: pGD-L; NPL: pGD-NPL containing tandem expression cassettes of N, P and L. pGD vectors expressing BSMV γb, TBSV p19 and TEV P1/HC-Pro RNA suppressors were also included in the mixture

^c^Systemic infections were verified by visual inspection for systemic symptoms and RT-PCR. The total percentage of systemic infectivity was calculated from independent experiments (Exp.)

Our previous studies showed that combined expression of the N, P, and L core protein in a single plasmid, when supplemented by additional amounts of L proteins (NPL + L), significantly improved SYNV rescue efficiency [16]. In our current experiments, the more desirable core protein mixture (NPL + L) also resulted in an increased infection rate in 35St-SYNV infiltrated plants (~ 20%). Nevertheless, none of the 121 plants infiltrated with the 35S-HH1-SYNV developed systemic infections (Table 1). These data show that capped SYNV agRNA transcripts produced by 35St promoter is substantially more efficient in supporting MR reporter gene expression and full-length virus rescue than the uncapped transcripts produced by HH1 Rz cleavage.

### Improved SYNV minireplicon efficiency with optimized hammerhead ribozymes

**Fig. 2:**
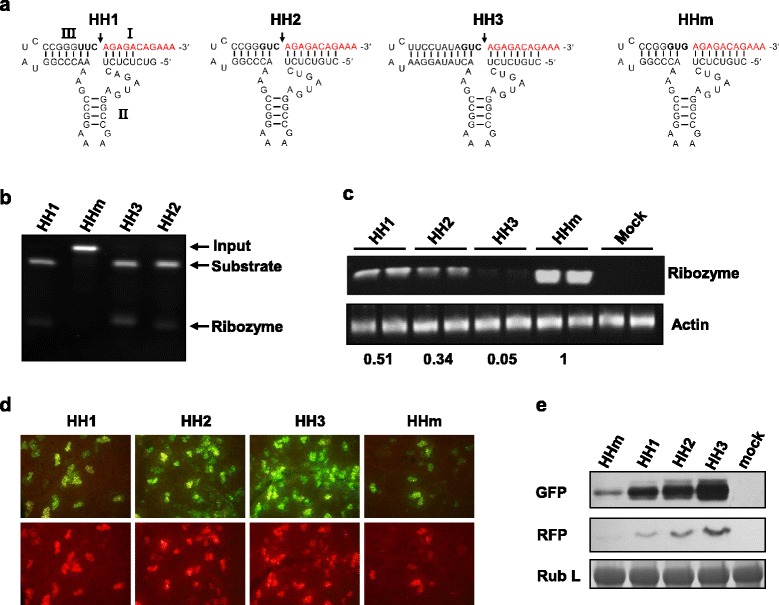
Effects of HH Rz optimization on minireplicon reporter gene expression. **a** Sequences and predicted RNA secondary structures formed by HH Rz variants preceding the 5′ termini SYNV agRNA sequence. The cleavage triplets in HH1, HH2 and HH3, as well as the inactive GUG triplet in HHm are shown in bold letters, and the 5′ termini of SYNV leader are in red. Arrows indicate cleavage site, and the three stem structures (I, II and III) are shown in the HH1 Rz. **b** Analysis of RNA cleavage in vitro by three intact HH Rzs and a mutant version (HHm). The cleavage products were resolved on a polyacrylamide gel and visualized after ethidium bromide staining. All three HH Rzs efficiently cleaved the fused SYNV RNAs, resulting in disappearance of input RNAs and concomitant release of substrate strands and short ribozyme RNAs. **c** Semi-quantitative RT-PCR analysis of ribozyme cleavage efficiency in vivo. The MR agRNAs with 5′ terminus fusions to the HH Rz variants were produced in plant tissues via agroinfiltration mediated expression. The uncleaved transcripts were detected by RT-PCR after 28 cycles of amplification using primers annealing to the ribozyme and the SYNV 5′ termini sequence. Actin cDNA amplification products were used as a control. **d** and **e** Improved MR reporter expression activity by using optimized HH Rzs. *N. benthamiana* leaves were agroinfiltrated with MR constructs containing the indicated HH Rz variants and necessary supporting plasmids as described in Fig. 1 legends. Fluorescent foci were monitored under fluorescence microscope at 9 dpi (**d**) and the expression levels of GFP and RFP were evaluated by western blot analysis (**e**). Coomassie blue-stained Rubisco large subunit (Rub L) is shown to indicate equal protein loading

We hypothesized that the HH1 Rz may have not efficiently processed the SYNV RNA 5′ terminus, and that the poor efficiency may have resulted in a portion of viral agRNAs with extra 5′ overhanging nucleotides that hindered recognition of the SYNV terminal *cis*-elements by the core proteins and subsequent nucleocapsid assembly. We examined the HH1 Rz-substrate strand secondary structure and revealed that this ribozyme contains a UUC cleavage triplet (Fig. 2a), which is less active than variants with a GUC triplet [19]. Therefore, we constructed a modified ribozyme version (HH2 Rz) with GUC as the cleavage triplet. In addition, the base paring between the 5′ end ribozyme sequence and the SYNV terminal leader (forming stem I) was extended from 5 base pairs (bps) in HH1 Rz to 8 bps in HH2 Rz to stabilize the ribozyme structure (Fig. 2a). We also designed another HH Rz variant (HH3 Rz) based on HH2 Rz. The HH3 Rz contains extended base pairing in the stem III, which has been shown to improve cleavage efficiency [17]. As a negative control, an HH Rz mutant (HHm Rz) with a catalytically inactive GUG triplet was also constructed (Fig. 2a). These modified HH Rz variants were incorporated into the 35S-HH1-MR plasmid to replace the HH1 Rz, resulting in 35S-HH2-MR, 35S-HH3-MR and 35S-HHm-MR plasmids. To compare the cleavage efficiencies of these HH Rz variants in vitro, RNA transcripts corresponding to the ribozyme and a short fragment of the SYNV terminal leader RNA were synthesized in vitro by T7 RNA polymerase and incubated under conditions permitting ribozyme autolysis. We found that all three HH Rz variants efficiently processed the substrate RNAs, and as anticipated, the inactive HHm Rz failed to release the fused substrate (Fig. 2b). To further assess their processing efficiencies of MR agRNA in vivo, the MR transcription plasmids containing different versions of HH Rz were agroinfiltrated into *N. benthamiana* tissues, and transcript cleavage was analysed by semi-quantitative RT-PCR. To determine the levels of uncleaved MR transcripts, we used a forward primer annealing to the 5′ end conserved HH sequence and a reverse primer corresponding to the SYNV leader RNA. As shown in Fig. 2c, the HH2-mediated cleavage was more efficient than that of the HH1, whereas the HH3 led to nearly complete substrate cleavage, and the HHm probably resulted in only negligible cleavage. Consistent with the in vivo cleavage data, when the MR transcription plasmids were co-delivered into *N. benthamiana* leaves together with the supporting plasmids, the optimized HH Rzs, especially HH3, substantially improved expression of MR fluorescent reporter genes (Fig. 2d and e). It is worth noting that the inactive HHm Rz did not abolish MR reporter gene expression, although the numbers of fluorescing cells and amounts of reporter protein recovered from the leaves were greatly reduced (Fig. 2d and e). These results suggest that an authentic viral 5′ terminus is important, but not absolutely required for MR agRNA activity. In summary, these experiments led to improved MR activity by using optimized Rz variants.

### Effects of the hammerhead ribozyme-processed SYNV agRNAs on full-length virus rescue

**Fig. 3:**
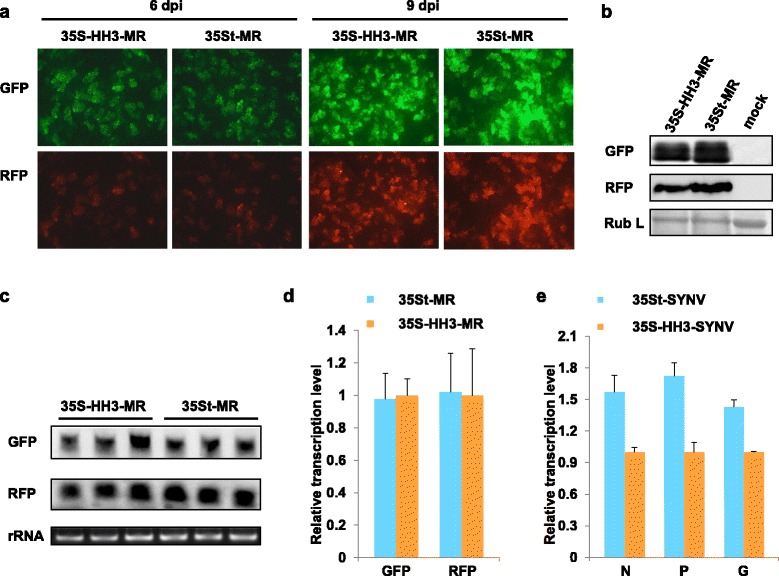
Efficiency of capped and HH3 Rz-processed agRNAs in supporting MR reporter gene expression. **a** Visualization of cells expressing GFP and RFP in *N. benthamiana* leaves infiltrated with agrobacteria harbouring 35S-HH3-MR or 35St-MR along with equal mixtures carrying core protein and VSR supporting plasmids. Fluorescent reporter expression was monitored at 6 and 9 dpi with an epifluorescence microscope. **b** Total protein extracts prepared from infiltrated leaves at 9 dpi were analysed in Western blots using anti-GFP and anti-RFP antibodies. Coomassie blue staining of the large subunit of Rubisco (Rub L) was used as loading control. **c** GFP and RFP mRNAs in infiltrated leaf tissues at 9 dpi were analysed by Northern blotting using probes against GFP and RFP sequences. Ethidium bromide staining of 25S ribosomal RNA (rRNA) was used as RNA loading control. **d** and **e** The transcript levels of MR and full-length agRNAs were analysed by qRT-PCR. Total RNA samples were isolated from *N. benthamiana* leaves infiltrated with 35S-HH3-MR or 35St-MR (**d**), or 35S-HH3-SYNV or 35St-SYNV (**e**), together with VSR mixtures. Two pairs of primers specific for GFP and RFP were used to amplify MR cDNAs, while three pairs of N, P and G specific primers were used to amplify SYNV full-length cDNA

Because the improved HH3 Rz resulted in nearly complete release of authentic viral 5′ terminus in vivo (Fig. 2c), we anticipated that it would support MR reporter gene expression as efficiently as 35St-transcribed agRNA. Indeed, under condition permissive for MR gene expression, *N.benthamiana* leaf patches infiltrated with 35S-HH3-MR and 35St-MR displayed similar numbers of GFP and RFP fluorescent foci at 6 and 9 days post infiltration (dpi) (Fig. 3a). Western blot (Fig. 3b) and Northern blot (Fig. 3c) analyses also confirmed comparable levels of reporter gene expression. We next substituted the HH3 Rz into the SYNV full-length cDNA clone to produce 35S-HH3-SYNV, and then tested the infectivity of this derivative under infiltration conditions (NPL + L) for optimum core protein expression. To our surprise, the 35S-HH3-SYNV barely supported SYNV systemic infection, and resulted in only one infection amongst 119 infiltrated plants, which is substantially less efficient that the ~20% systemic infection rate observed in 35St-SYVN infiltrated plants (Table 1). Because RNA capping provides protection against RNase degradation, it is possible that 35St transcribed SYNV agRNA derivatives may accumulate to higher levels than those processed by HH Rzs, thereby increasing rescue efficiency. We thus compared the steady state levels of SYNV MR and full-length agRNA transcripts produced by 35St and 35S-HH3. In these experiments, qRT-PCR analyses with GFP- and RFP-specific primer pairs indicated that similar amounts of MR transcripts accumulated in leaves infiltrated with 35St-MR and 35S-HH3-MR (Fig. 3d). Indeed, in the case of SYNV full-length agRNA, the accumulation of the capped transcripts produced by 35St were slightly higher than the uncapped, HH3 Rz-processed transcripts (1.4- to 1.7-fold increase depending on whether the N, P or G-specific primers were used for detection, Fig. 3e). However, the moderate increase in transcript levels is unlikely to be solely responsible for the markedly improved infectivity of the capped SYNV agRNA, so we postulated that additional function(s) may be provided by the cap structure during the course of full-length virus rescue.

### Possible role of N protein expressed *in cis* from capped SYNV agRNA in recovery of full-length virus

**Fig. 4:**
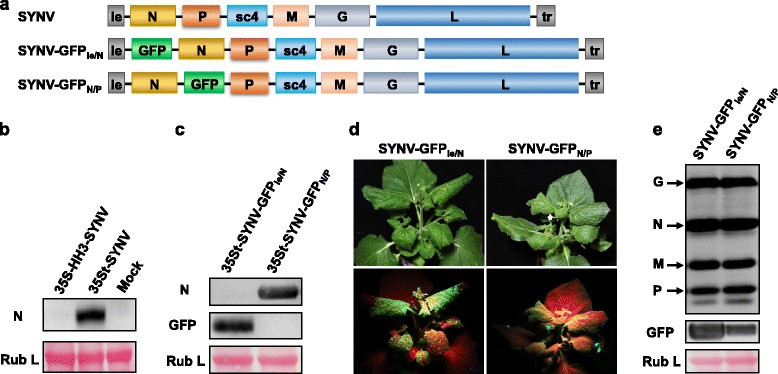
Function of N proteins translated *in cis* from capped agRNA in SYNV rescue. **a** Diagram showing insertion of the GFP gene between the leader and N genes (SYNV-GFP_le/N_) or between the N and P genes (SYNV-GFP_N/P_) in SYNV agRNAs. GFP mRNA transcription was directed by a duplicated leader/N or N/P gene junction sequences, in SYNV-GFP_le/N_ or SYNV-GFP_N/P_, respectively. **b** and **c** Transient expression of genes located at the 5′ termini of SYNV agRNAs. Total protein samples were exacted from *N. benthamiana* leaves at 5 dpi after agroinfiltration with 35S-HH3-SYNV and 35St-SYNV (**b**), or 35St-SYNV-GFP_le/N_ and 35St-SYNV-GFP_N/P_ (**c**). *Agrobacteria* carrying VSR plasmids were also included in the infiltration mixtures to suppress RNA silencing. Protein gels were blotted with anti-N and anti-GFP antibodies. **d** Symptoms of recombinant SYNV-GFP_le/N_- and SYNV-GFP_N/P_-infected plants. Recombinant viruses recovered from *N. benthamiana* plants were passaged to healthy plants by sap inoculation. The infected plants were photographed at 16 dpi under visible light (upper panels) and ultraviolet (UV) light (bottom panels). **e** Immunoblot analysis of SYNV structural proteins and GFP expression in systemically infected tissues of plants inoculated with recombinant SYNV-GFP_le/N_ and SYNV-GFP_N/P_ using antibodies against disrupted SYNV virions or GFP. In (**b**), (**c**) and (**e**), the protein blots were stained with Ponceau S to verify similar level of the large subunit of Rubisco (Rub L)

One important function of the mRNA cap is to recruit the eIF4F cap binding complex during translation initiation [20]. Indeed, the 35St-directed synthesis of capped SYNV MR agRNA allows direct translation of the GFP reporter gene located at the N-terminus (Fig. 1c). We also observed that abundant N protein expression occurs in leaf tissues infiltrated with agrobacteria harbouring the 35St-SYNV plasmid, but similar expression was not detected after agroinfiltration of the 35S-HH3-SYNV plasmid derivative (Fig. 4b). If the *in cis* translated N protein plays a role during the recovery of SYNV from cDNA plasmids, we reasoned that abrogating N protein transient expression from agRNA transcripts would reduce the rescue efficiency. For this purpose, we engineered an SYNV full-length agRNA derivative (SYNV-GFP_le/N_) that contains a GFP gene insertion in the N ORF position, followed by a duplicated gene junction sequence to direct the N mRNA synthesis (Fig. 4a). In this configuration, the N ORF was shifted to an internal position in the agRNA transcripts downstream of the 5′ GFP ORF, which should prevent the N gene from being translated by eukaryotic ribosomes. As a control, we used our previously generated SYNV-GFP_N/P_ [16], in which the GFP gene was placed between the N and P transcription units. When the SYNV-GFP_le/N_ agRNAs were transcribed in agroinfiltrated plant cells under the control of the 35St promoter (35St-SYNV-GFP_le/N_), N protein expression was abolished as we anticipated, and GFP expression was readily detected by Western blotting (Fig. 4c). In contrast, SYNV-GFP_N/P_ agRNA transcription resulted in abundant N protein expression, but not GFP expression (Fig. 4c). When assayed for systemic infectivity in the presence of core proteins (NPL + L) and VSRs plasmids, the 35St-SYNV-GFP_le/N_ had greatly reduced infection rates compared to similar 35St-SYNV-GFP_N/P_ infiltrations (4.4% versus 21.9%, Table 1). To exclude the possibility that differential infectivity was due to an inherent virulence of the recombinant viruses, the progenies of the recovered viruses were passaged to healthy plants by mechanical sap inoculation, and their infection dynamics were compared. Both SYNV-GFP_le/N_ and SYNV-GFP_N/P_ initiated systemic infection at about 13 dpi and ultimately led to indistinguishable symptoms in 100% of the inoculated plants (Fig. 4d, data not shown). Western blot analyses also confirmed similar levels of SYNV structural protein accumulation in tissues of systemic infected plants (Fig. 4e). Note that more abundant GFP expression was present in SYNV-GFP_le/N_ infected tissues than in SYNV-GFP_N/P_ tissues as revealed by fluorescence photography (Fig. 4d) and Western blotting (Fig. 4e), which is consistent with polar transcription of mRNAs observed in mononegaviruses [21]. These data suggest that capped agRNA-mediated transient expression of the N protein correlates with high rescue efficiency of recombinant SYNV, but it is unclear whether the *in cis* expressed N proteins *per se* are responsible for the increased infectivity, or simply the cumulative higher proportions of the total N proteins (the *in cis* expressed N proteins together with the N proteins provided *in trans* from co-delivered supporting plasmids) enhances the initial stages of infection. To discriminate between these two possibilities, we infiltrated *N. benthamiana* plants with an *Agrobacterium* mixture carrying the 35S-HH3-SYNV (which is unable to express the N protein *in cis*) and with the (NPL + L + N) core protein plasmids to supply extra amounts of the N proteins *in trans*. Under these conditions, none of the 72 plants infiltrated with the NPL + L + N mixture developed systemic infections (Table 1). Taken together, these data suggest that a specific function(s) of the *in cis* expressed N proteins from capped agRNA transcripts is needed for efficient SYNV rescue.

## Discussion

Current SYNV reverse genetic systems rely on Pol II (CaMV 35S) promoter-based intercellular transcription of viral agRNAs and core protein mRNAs through agroinfiltration [7, 15, 16]. Our initial SYNV MR system employed HH Rz (HH1) and HDV Rz to process the viral termini [15], which resembles the strategies used in various Pol II-based rescue systems of animal NSVs. In this study, we show that MR reporter gene expression can be substantially improved by using more active HH Rz variants to generate the exact agRNA 5′ termini. This finding, together with several animal NSVs rescue studies, confirmed the importance of availability of exact 5′ viral ends for efficient NSV rescues, and the necessity of using highly active ribozymes for viral RNA processing [8–10, 22]. For example, in the case of Rabies virus (a member in the genus *Lyssavirus*, family *Rhabdoviridae*), optimization of the 5′ HH Rz and 3′ HDV Rz leads to increased MR reporter gene expression by 10-fold and virus rescue efficiency by more than 100-fold [10]. On the other hand, we also should note when the catalytic inactive HHm Rz was used in our study, SYNV MR reporter gene expression was reduced but not completely abolished. The inactivation of the HH Rz would result in synthesis of SYNV agRNAs with a 5′ extension of 47-nucleotides of the ribozyme sequence plus a short stretch of promoter sequence downstream of the transcription start site. Nevertheless, the presence of residual reporter gene expression with the HHm Rz MR construct suggests that the SYNV L proteins are able to initiate mRNA synthesis from internally localized promoter signals, albeit with reduced efficiency.

Although the optimized HH Rz gave rise to agRNA derivatives that are able to support high levels of SYNV MR reporter gene expression, rescue of recombinant SYNV using the 5′ ribozyme processing strategy turned out to be very inefficient. In comparison, the capped agRNAs precisely transcribed by 35St achieved productive recombinant virus recovery rates. The 35St promoter was originally designed to produce capped, 5′ terminal precise gRNA transcripts from delivered cDNA clones of plant positive-stranded RNA viruses [18]. In this case, the 5′ cap group functions to facilitate translation of viral gRNA transcripts to produce viral replication-related proteins that subsequently start infection cycles. However, during natural NSV infections, agRNAs are believed not to act as translation templates, because they are (i) uncapped and nonpolyadenylated, (ii) always encapsidated by their N or NP proteins and never exist in a free form, and (iii) sequestered in the viroplasm. This is certainly not the case with plasmid-based agRNA transcripts produced during NSV reverse genetics experiments. In this study we have demonstrated that the capped SYNV agRNA transcripts mediate efficient translation of genes located at the 5′ proximal termini of the agRNAs, e.g. the N genes. Furthermore, the ability of the agRNAs to express the N proteins correlates with high rescue efficiencies, and supplying extra amounts of N proteins *in trans* from plasmids did not improve the rescue of an agRNA derivative that are unable to produce the N proteins. Based on these data, we suggest a *cis*-preferential encapsidation model to explain the superior activity of capped agRNAs during SYNV rescue. The SYNV N proteins may efficiently bind *in cis* to the capped agRNAs from which the N proteins are derived. Through protein-protein interactions, these preassembled N-agRNA complexes subsequently recruit additional core proteins (N, P, and L proteins) expressed from co-delivered supporting plasmids to facilitate the assembly of functional nucleocapsids. In contrast, nucleocapsid assembly with HH Rz-processed agRNAs may be less efficient, because core proteins expressed *in trans* from provided plasmids may not be located in close proximity for effective encapsidation. It is worth noting that despite of the facilitating role of the *in cis* expressed N proteins, N supporting plasmid is still needed for recovery of recombinant SYNV as shown in our previous study [16]. Therefore, it appears that the agRNAs-mediated expressions of N proteins are present below the threshold level required for assembly of biologically active nucleocapsids.

It is intriguing to ask whether the proposed *cis*-acting function(s) of N protein also occurred in other NSV rescue systems where viral agRNAs were transcribed for in vivo nucleocapsid reconstitution. Notably, many studies have previously shown that T7 Pol-generated agRNAs transcripts derived from a number of nonsegmented or segmented animal NSVs are able to produce viral protein(s) encoded in the 5′ terminal ORFs of agRNAs, e.g. the N proteins, and both the N and L proteins, for nonsegemented and segmented NSVs, respectively [23–31]. More importantly, these *in cis* expressed viral core proteins are sufficient to support virus rescue in the absence of their respective supporting protein expression plasmid(s). This was first documented for two mononegaviruses, the human parainfluenza viruses-3 (in the *Paramyxoviridae* family) [28] and a fish novirhabdovirus [24]. In these cases, rescue was achieved by co-expression of the P and L proteins from transfected supporting plasmids along with the T7 transcripts of agRNA, although providing the N-expressing plasmids increased the rescue efficiency. With regard to several members of the bi-segmented *Arenaviridae* and tri-segmented *Bunyaviridae*, recombinant viruses are generated by transfection of just the agRNA transcription plasmids [24–27, 29–31], indicating that the two required core proteins, the N (or NP) and L proteins, are both translated from the primary agRNA transcripts. Interestingly, for the orthobunyavirus La Crosse virus, supplying N and L proteins *in trans* from plasmids even abrogated the rescue [25]. These discrepancies in requirement of core protein supporting plasmids may be due to varied levels of the N and/or L proteins expressed from primary agRNA transcripts derived from different viruses. To our best knowledge, the supporting plasmids-independent rescues have only been documented in T7 Pol-based systems, in which cytoplasmic transcription of agRNAs may allow translation to occur. In addition, expression of T7 Pol in mammalian cells is commonly achieved by infection with a recombinant vaccinia virus, which encodes enzymes that non-specifically cap cytoplasmic RNAs [32, 33], so it is possible that a portion of agRNAs generated in those reverse genetics systems are actually capped. Overall, the above mentioned studies indicate that agRNA transcripts for various animal NSVs permit abundant expression of N proteins (as well as L proteins in the case of arenaviruses and bunyaviruses), which in turn recruit the agRNAs for nucleocapsid assembly. However, it remains to determine whether agRNAs that are able to translate core proteins support higher rescue efficiencies than untranslatable agRNAs.

## Conclusions

In summary, we have compared two strategies used to generate exact viral 5′ terminus of SYNV agRNA derivatives for their efficiencies in MR reporter gene expression and virus rescue. Our study shows that the capped agRNAs are superior to the uncapped, HH ribozyme-processed agRNAs, and indicates that the *cis*-acting N protein expressed from the capped agRNA functions to facilitate recovery of SYNV from cloned cDNAs. Our results also show that conditions for optimized rescue of the truncated MR may need to be modified when devising rescue strategies for full-length recombinant viruses.

## Additional file

Additional file 1:
**Table** **S1.** List of primers used in this study. (DOCX 18 kb)

## Abbreviations

35St: Truncated 35S promoter
agRNA: Antigenomic RNA
CaMV: Cauliflower mosaic virus
cDNA: complementary DNA
Dpi: Days postinfiltration
GFP: Green fluorescence protein
gRNA: Genomic RNA
HDV Rz: Hepatitis delta virus ribozyme
HH Rz: Hammerhead ribozyme
L: large RNA-Dependent RNA polymerase
MR: Minireplicon
N: Nucleoprotein
*N.benthamiana*: *Nicotiana benthamiana*
NSV: Negative-stranded RNA virus
OD: Optical density
P: Phosphoprotein
Pol I: RNA polymerase I
Pol II: RNA polymerase II
qRT-PCR: Quantitative reverse transcription PCR
RFP: Red fluorescence protein
SYNV: Sonchus yellow net rhabdovirus
T7 Pol: T7 RNA polymerase
UTR: Untranslated region
UV: Ultraviolent
VSR: Viral suppressor of RNA silencing
