# Supplementary material for: Capped antigenomic RNA transcript facilitates rescue of a plant rhabdovirus
Source: Virol J. 2017 Jun 13;14:113. doi: 10.1186/s12985-017-0776-7 (PMC5470278; doi:10.1186/s12985-017-0776-7)
Supplement: Additional file 1: — Table S1. List of primers used in this study. (DOCX 18 kb) [file 12985_2017_776_MOESM1_ESM.docx]

Supplementary Table 1. List of primers used in this study.

| Primer name | Primer sequences (5’-3’)^a^ |
| --- | --- |
| T-DNA LB *Spe*I *Pvu*I R | cgca**actagt**gggaaggg**cgatcg**gtgc |
| T-DNA RB F | cgctcttttctcttaggtttac |
| pCambia HRz R1 | gcctttcggcctcatcagagagacaggggaagatccaggcctct |
| HRz *Xma*I R2 | ctga**cccggg**ataccgggtttcggcctttcggcctcatcag |
| SYNV(+)1 F | agagacagaaactcagaaaatacaatca |
| TC+ SYNV(+)1 *Xma*I F | tat**cccggg**tcagagacagaaactcagaaaatacaatc |
| TG+ SYNV(+)1 *Xma*I F | tat**cccggg**tgagagacagaaactcagaaaatacaatc |
| SYNV HRz_3_ F | aggaaaggaattcctatagtcagagacagaaactcag |
| SYVV HRz_3_ R | aggaattcctttcctatagtttcggcctttcggcctcat |
| pCB301 *Not*I F | acaacgccg**gcggccgc**ggtgt |
| pCB301 *Nco*I R | ggatcgattt**ccatgg**cctagacaaat |
| N 5' UTR F | tattttttgagtattttaattgcaggt |
| N 5' UTR R | aatactcaaaaaatacgatagtatgagat |
| SYNV N P junction R | ggcctagacaaataatacaaacagacaaat |
| P 5' UTR-N F | ttatttgtctaggccatgagcactacaccaacaat |
| T7 HH MR F | taatacgactcactatagggctgtctctctgatgaggccgaaaggc |
| MR HR SqRT-PCR F | gtctctctgatgaggccgaaaggc |
| MR HR SqRT-PCR R | tgctcaccatgctagcgattacct |
| eGFP F | atggtgagcaagggcgagga |
| eGFP R | ttacttgtacagctcgtccatgccga |
| DsRed F | atggcctcctccgagaacgt |
| DsRed R | ttataggaacaggtggtggcggccct |
| Actin qPCR F | caatccagacactgtactttctctc |
| Actin qPCR R | aagctgcaggtatccatgagacta |
| N qPCR F | gttcagtgatgcagtcaagtatcctat |
| N qPCR R | gtcagtacttggtaccagctgtgttata |
| P qPCR F | aagtattcagctccacaatactacatctga |
| P qPCR R | agatcaccattatcccctatgtcttgat |
| G qPCR F | ccagaatggatcaaaggttcagttc |
| G qPCR R | ctcaccttccatagtacacttagcacaat |
| RFP qPCR F | gctccaaggtgtacgtgaagcac |
| RFP qPCR R | gcccatggtcttcttctgcat |
| GFP qPCR F | tggtgaaccgcatcgagct |
| GFP qPCR R | gtgctcaggtagtggttgtcg |

^a^ Restriction enzyme sites are shown in bold.
